# Preliminary characterization of N-trimethylchitosan as a nanocarrier for malarie vaccine

**DOI:** 10.1186/1758-2946-4-S1-P47

**Published:** 2012-05-01

**Authors:** Petra O Nnamani, Ngozi J Nwodo, Scoles Giacinto

**Affiliations:** 1 Drug Discovery and Drug Delivery Research Unit, Faculty of Pharmaceutical Sciences, University of Nigeria, Nsukka 410001, Enugu State, Nigeria; 2 Nanotechnology Unit, ICS-UNIDO International Center for High Technology and New Materials, AREA Science Park, Padriciano 99, Trieste, Italy

## 

Vaccination is considered to be most effective way of fighting infectious diseases like malaria etc [1]. N, N, N-trimethylchitosan (TMC) was synthesized from chitosan. Nanoparticles of the TMC were prepared in various media (milliQ water, Na_2_CO_3_ (pH 10.92), Na_2_HPO_4_ (pH 9.01) and alhydrogel^®^ beads which were characterized as adjuvant for possible vaccine delivery. The nanoparticles were analyzed using microscopy (Phase contrast microscope and Confocal laser scanning microscope), and Malvern zetasizer Nano- ZS. Time-resolved particle size analysis was performed after one month storage of the TMC nanoparticles at 4 °C. The result of the study showed that PBS was the best medium that produced cationic, monodispersed and stable TMC nanoparticles of less than 65 nm forming a compatibly homogeneous system even upon storage. Microscopy of the polyelectrolyte doped nanoparticles showed a clear coating due to PSS at the periphery of the particles and a fluorescent core with some tiny central hollow cavities Confocal microscopy of the alhydrogel beads showed particle size of 1.6 µm. The fluorescent dye (PSSRhodamine) coated the entire particle surface suggesting a more or less adsorption process for the antigen delivery [2]. Hence, the hope of nanocarrier for malaria vaccine.
